# Vagaries of Fluorochrome Reporter Gene Expression in Foxp3^+^ Regulatory T Cells

**DOI:** 10.1371/journal.pone.0041971

**Published:** 2012-08-06

**Authors:** Sonja Schallenberg, Cathleen Petzold, Pei-Yun Tsai, Tim Sparwasser, Karsten Kretschmer

**Affiliations:** 1 Immunotolerance in Regeneration, CRTD/DFG-Center for Regenerative Therapies Dresden, Technical University Dresden, Dresden, Germany; 2 Institute of Infection Immunology, TWINCORE/Centre for Experimental and Clinical Infection Research, Hannover, Germany; Institut Pasteur, France

## Abstract

CD4^+^CD25^+^ regulatory T (Treg) cell lineage commitment and expression of the transcription factor Foxp3 can be induced at the CD4^+^CD8^+^ double-positive (DP) and CD4^+^CD8^?^ single-positive stages of thymic development, as well as in postthymic CD4^+^ T cells in peripheral lymphoid tissues. The availability of transgenic mice with Foxp3-dependent fluorochrome reporter gene expression has greatly facilitated studies on the intra- and extrathymic generation of murine Foxp3^+^ Treg cells. Here, we performed a comparative analysis of thymic Treg cell development and peripheral compartments of mature Treg cells in various transgenic strains with gene targeted and bacterial artificial chromosome (BAC)-driven Foxp3-fluorochrome expression. These studies revealed a relative deficiency of Foxp3^+^ DP thymocytes selectively in mice with targeted insertion of the fluorochrome reporter gene coding sequence into the endogenous *Foxp3* gene. While Foxp3 BAC-driven fluorochrome expression in *ex vivo* CD4^+^ T cells was found to faithfully reflect Foxp3 protein expression, we provide evidence that Foxp3 BAC transgenesis can result in sizable populations of Foxp3^+^ Treg cells that lack fluorochrome reporter expression. This could be attributed to both timely delayed up-regulation of BAC expression in developing Treg cells and the accumulation of peripheral Foxp3^+^ Treg cells with continuous transcriptional inactivity of the Foxp3 BAC transgene.

## Introduction

A common hallmark of intra- and extrathymic CD4^+^CD25^+^ regulatory T (Treg) cell lineage commitment is the induction of Foxp3 expression as a consequence of appropriate T cell receptor (TCR) engagement with MHC class II:peptide ligands [1], resulting in stabilization and amplification of Treg cell-specific gene transcription [2], [3] through Foxp3 occupancy of key target gene promoters [4], [5].

In the thymus, analysis at the single-cell level provided evidence for the induction of Foxp3 expression at the CD4^+^CD8^+^ double-positive (DP) stage [6], [7], [8], [9] and a precursor-progeny relationship between DP and CD4^+^CD8^?^ single-positive (CD4SP) Foxp3^+^ thymocytes [8]. The prevailing view that thymic induction of Foxp3 expression occurs predominantly, if not exclusively, at the DP stage has been challenged by several observations, including the capacity of TCR transgenic thymocytes to initiate Antigen (Ag)-driven Foxp3^+^ Treg cell induction at the CD4SP stage [10] and the enrichment of precommitted immediate precursors to Foxp3^+^ Treg cells among CD25^+^Foxp3^?^ CD4SP thymocytes in non-TCR transgenic mice [11]. Collectively, these findings support a model of thymic Treg cell development, in which lineage commitment and subsequent induction of Foxp3 expression at both the DP and CD4SP stage can occur in parallel. However, the predominance of Foxp3^+^ CD4SP cells and the low proportional contribution of Foxp3^+^ DP cells to the overall population of Foxp3^+^ cells in the adult thymus suggests that induction of Foxp3 expression in DP cells represents a relatively rare occurrence [12], arguing for a minor role of Foxp3^+^ DP thymocytes in the generation of the peripheral Treg cell pool.

In peripheral lymphoid tissues, naïve CD4^+^Foxp3^?^ T cells can acquire a Foxp3^+^ Treg cell phenotype in a variety of experimental settings, such as lymphopenia-driven homeostatic expansion [13], [14], [15] and subimmunogenic administration of either free Ag [16], [17], [18], [19], [20] or DEC-205^+^ dendritic cell-targeted foreign [21], [22], [23], [24] and self-Ag [25], [26]. In addition, the existence of CD4^+^Foxp3^–^ precursors in lymph nodes (LNs) of non-TCR transgenic, non-manipulated mice that are precommitted to differentiate into Foxp3^+^ Treg cells has provided evidence on the relevance of peripheral Treg cell induction in the steady state [27]. Nevertheless, the contribution of thymic and extrathymic Treg cell developmental pathways to the phenotypic and functional heterogeneity of the peripheral Treg cell pool ([28]], and references therein) has remained difficult to establish by direct evidence [29], [30], [31], [32].

Fluorochrome reporter mice to track Foxp3 expression in single viable cells have greatly facilitated studies on the biology of murine Foxp3^+^ Treg cells. Several transgenic strategies have been employed to achieve co-expression of Foxp3 with fluorochromes. This includes ‘knock-in’ gene-targeting, designed to express a fluorochrome either as a fusion protein with Foxp3 [7] or from an internal ribosome entry site (IRES) downstream of the *Foxp3* coding region [6], [33], and transgenesis employing Foxp3 bacterial artificial chromosomes (BACs) that contain an inserted fluorochrome encoding gene [34], [35], [36]. Foxp3-dependent fluorochrome reporter mice have now become widely available to the scientific community and are commonly used to study the generation, life style and function of Foxp3^+^ Treg cells. Although of considerable interest, potential pitfalls inherent to transgenic approaches of Foxp3-dependent reporter gene expression have only recently begun to be explored [37], [38]. While transgenic fluorochrome expression as a Foxp3 fusion protein [7] or from an IRES [6], [33] assures accurate fluorochrome-based Foxp3 detection, this may not necessarily hold true for Foxp3 BAC-mediated fluorochrome expression, as endogenous Foxp3 and transgenic fluorochrome proteins are not physically linked. In addition, targeted insertion of a complete fluorochrome coding sequence, either into the endogenous *Foxp3* gene locus or the transgenic Foxp3 BAC, poses the risk of aberrations in gene expression, e.g. by interference with essential regulatory DNA elements within the targeted *Foxp3* gene locus.

Here, we have analyzed Foxp3-dependent fluorochrome expression in developing thymocytes and in peripheral compartments of mature Treg cells, comparing various gene targeted and BAC transgenic Foxp3-fluorochrome reporter strains. These studies revealed a deficiency in green fluorescent protein (GFP) expression selectively in DP thymocytes of Foxp3^GFP^ mice. In mice expressing a Cre recombinase (Cre)-GFP fusion protein as a Foxp3 BAC transgene (here referred to as BAC-Foxp3^Cre-GFP^ mice; [35]), we identified sizable populations of Foxp3^+^ cells in both thymus and peripheral lymphoid tissues that lack GFP expression. While the existence of Foxp3^+^GFP^−^ cells in the thymus of BAC-Foxp3^Cre-GFP^ mice could be readily explained by timely delayed up-regulation of GFP expression in Foxp3^+^ Treg cell lineage committed thymocytes, we provide evidence that the peripheral population of Foxp3^+^GFP^−^ Treg cells lacks BAC-Foxp3^Cre-GFP^ activity throughout their entire development and lifespan.

## Materials and Methods

### Mice

Foxp3^GFP^ mice [9] were crossed to the BALB/c or C57BL/6 genetic background for at least twelve generations. Foxp3^IRES-GFP^ mice were on the BALB/c genetic background ([33]; The Jackson Laboratory). BAC-Foxp3^Cre-GFP^ mice ([35]; The Jackson Laboratory) and Foxp3 BAC transgenic mice expressing a human diphtheria toxin receptor-GFP fusion protein (termed ‘depletion of regulatory T cell’ mice, DEREG; here referred to as BAC-Foxp3^DTR-GFP^ mice; [34] were on the NOD genetic background. As indicated, BAC-Foxp3^Cre-GFP^ mice were additionally bred to mice, which expressed a Cre recombination reporter allele of the ubiquitously expressed ROSA26 locus containing a loxP site–flanked STOP cassette, followed by a DNA sequence encoding yellow fluorescent protein (YFP; R26Y) [39] to obtain BAC-Foxp3^Cre-GFP^ × R26Y mice. Mice were housed and bred at the Experimental Center of the Medizinisch-Theoretisches Zentrum (Dresden University of Technology, Germany) under specific pathogen-free conditions. All animal studies were performed in strict accordance with German Animal Welfare legislation. All protocols were approved by the Institutional Animal Welfare Officer (Tierschutzbeauftragter), and necessary licenses were obtained from the regional license granting body (Landesdirektion Dresden, Germany; permit numbers: 24-9168.11-1/2008-12).

### Flow Cytometry and Cell Sorting

Single cell suspensions of thymus, spleen, or a pool of subcutaneous LNs (scLNs; *Lnn. mandibularis, Lnn. cervicales superficiales, Lnn. axillares et cubiti, Lnn. inguinales superficiales,* and *Lnn. subiliaci*) were prepared using 70 µm cell strainers (BD). Monoclonal antibodies (mAbs) to CD4 (RM4-5), CD8 (53–6.7), and CD25 (PC61, 7D4) as well as Pacific Blue- and PE-conjugated streptavidin were obtained from eBioscience or BD. Before FACS, for some experiments, CD25^+^ cells were enriched from single cell suspensions using biotinylated antibodies directed against CD25, streptavidin-conjugated microbeads, and the AutoMACS magnetic cell separation system (Miltenyi Biotec). Where indicated, intracellular Foxp3 expression was analyzed using the mAb FJK-16s (eBioscience) and the Foxp3 staining buffer set (eBioscience) according to the manufacturer’s protocol. Before the analysis of Foxp3 protein expression using mAbs, GFP/YFP-expressing cells were isolated by flow cytometry to high purity and subsequently subjected to intracellular staining (ICS), as the GFP/YFP fluorescence was found to be severely abrogated by the fixation/permeabilization procedure. Samples were analyzed on an LSRII or FACSCalibur or sorted using a FACS Aria II (BD). Data were analyzed using FlowJo software (Tree Star, Inc.).

### Gene Expression Analysis

Total RNA was extracted from FACS purified T cell populations using the RNeasy Mini kit and DNase I digestion (QIAGEN), and cDNA was synthesized according to the manufacturer’s recommendations (SuperScript II reverse transcriptase; Invitrogen). The QuantiFast SYBR Green PCR kit (QIAGEN) and a Mastercycler ep realplex thermal cycler (Eppendorf) were used to analyze cDNA in replicates, as indicated. The following primers were used: β-actin, 5′-TGGAATCCTGTGGCATCCATGAAAC-3′ and 5′TAAAACGCAGCTCAGTAACA GTCCG-3′; Foxp3, 5′-CCCAGGAAAGACAGCAACCTT-3′ and 5′-CAAACAGGCC GCCGTCTGGAGCC-3′; EGFP, 5′-CTGACCTACGGCGTGCAGTGCTTCAG-3′ and 5′-GTGCTCAGGTAGTGGTTGTCGG-3′ and EYFP, 5′-CATCTGCACCACC GGCAAG-3′ and 5′-GCGAAGCACTGCAGGCCGTAGCCGAA-3′.

### Statistical Analysis

The one-way ANOVA with Bonferroni’s multiple comparison post test was performed using the Prism 5.01 software (GraphPad Software). Differences were considered as significant when * p < 0.05, or *** p < 0.001.

## Results

### Foxp3 Reporter Gene Expression during Thymic Treg Cell Development

**Figure 1:**
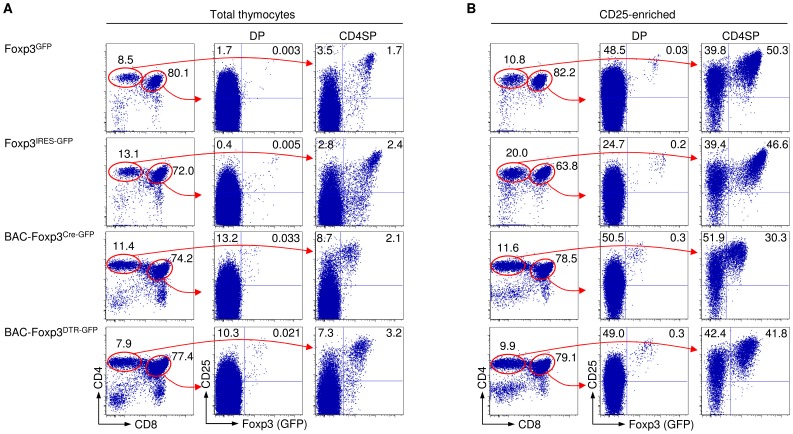
Tracking Foxp3-dependent GFP expression during thymic Treg cell development. Representative flow cytometry of GFP and CD25 expression among DP and CD4SP thymocytes of Foxp3^GFP^, Foxp3^IRES-GFP^, BAC-Foxp3^Cre-GFP^ and BAC-Foxp3^DTR-GFP^ mice, as indicated, (**A**) before and (**B**) after magnetic bead enrichment of CD25^+^ cells. All mice were six weeks old. Lines with arrowheads illustrate the gating scheme. Numbers in dot plots indicate percentages of cells in the respective quadrant or gate.

Flow cytometric analysis of total thymocyte populations suggested that the population size of DP cells with Foxp3-dependent GFP expression in Foxp3^GFP^ mice is near the detection limit of the method (Fig. 1A, top). Concurrent analysis of age-matched Foxp3^IRES-GFP^, BAC-Foxp3^Cre-GFP^ and BAC-Foxp3^DTR-GFP^ mice indicated the existence of a small, albeit clearly discernable population of GFP^+^ DP cells, with the vast majority expressing high levels of CD25 (Fig. 1A). CD25 bead enrichment of total thymocytes prior to GFP expression analysis allowed us to visualize a minute Foxp3^GFP+^CD25^+^ DP cell population (Fig. 1B, top), although their proportional underrepresentation relative to Foxp3^IRES-GFP^, BAC-Foxp3^Cre-GFP^ and BAC-Foxp3^DTR-GFP^ mice was maintained (Fig. 1B).

**Figure 2:**
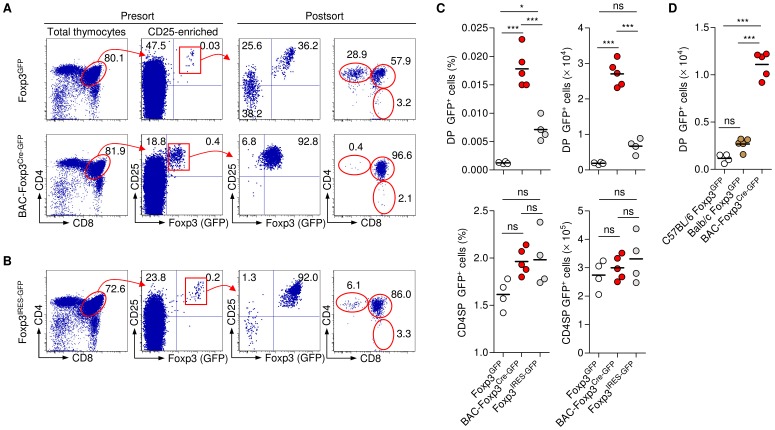
Comparative quantification of GFP^+^ DP cells. Flow cytometric isolation of GFP^+^ DP thymocytes from (**A**) Foxp3^GFP^, BAC-Foxp3^Cre-GFP^ and (**B**) Foxp3^IRES-GFP^ mice. Representative dot plots (from left to right) show presort analysis of CD4/CD8 expression among total thymocytes and CD25/GFP expression among CD25-enriched populations of gated DP cells, as well as postsort analysis of CD25/GFP and CD4/CD8 expression after flow cytometric isolation according to sort gates for CD25-enriched CD25^+^GFP^+^ cells, as indicated. Lines with arrowheads illustrate the gating scheme. Numbers in dot plots indicate percentages of cells in the respective quadrant or gate. (**C**) Quantification of GFP^+^ thymocytes. Percentages (left) and numbers (right) of GFP^+^ DP cells (top) and GFP^+^ CD4SP cells (bottom) from indicated Foxp3 reporter strains, revealed after postsort analysis as depicted in (**A,B**). All mice were six weeks old. (**D**) Numbers of GFP^+^ DP thymocytes from eleven-week-old Foxp3^GFP^ mice on the C57BL/6 (left) and BALB/c (middle) genetic background, as compared to age-matched BAC-Foxp3^Cre-GFP^ mice (right). Dots and horizontal lines represent individual mice and mean values, respectively. * p < 0.05, *** p < 0.001, ns, non-significant (one-way ANOVA with Bonferroni’s multiple comparison post test).

In addition to exceedingly low cell numbers, and in agreement with previous reports [12], [27], our attempts to FACS purify Foxp3^+^CD25^+^ DP thymocytes from Foxp3^GFP^ mice was hampered by a propensity of GFP^?^ DP cells to form doublets with GFP^+^ CD4SP cells, as revealed by post sort analysis of CD4, CD8 and GFP expression (Fig. 2A, top). In contrast, flow cytometric isolation yielded pure populations of GFP^+^CD25^+^ DP cells from thymi of BAC-Foxp3^Cre-GFP^ (Fig. 2A, bottom), Foxp3^IRES-GFP^ (Fig. 2B) and BAC-Foxp3^DTR-GFP^ (data not shown) mice. To minimize the inclusion of doublets, we based our quantification of GFP^+^ thymocytes on the postsort analysis, after CD25 bead enrichment and flow cytometric isolation using stringent gating criteria. These experiments consistently revealed an approximately ten-fold increase in the population size of GFP^+^ DP thymocytes (Fig. 2C, top) in BAC-Foxp3^Cre-GFP^ mice (0.019±0.006%; 2.05±0.24×10^4^ cells), as compared to Foxp3^GFP^ mice (0.0012±0.0002%; 0.19±0.03×10^4^ cells). Similar results were obtained with GFP^+^ DP cells from age-matched Foxp3^GFP^ mice on the C57BL/6 and BALB/c genetic backgrounds (Fig. 2D). In contrast to Foxp3^GFP^ mice, significant populations of GFP^+^CD25^+^ DP cells were detectable in Foxp3^IRES-GFP^ mice, although percentages and numbers failed to reach levels observed in BAC-Foxp3^Cre-GFP^ mice (Fig. 2C, top). Thus, it appears that, in comparison with Foxp3 BAC transgenesis, the transgenic expression of GFP both as a Foxp3 fusion protein and from an IRES downstream of the *Foxp3* gene results in considerably reduced Foxp3^+^CD25^+^ DP compartment sizes, albeit to different degrees. Importantly, the size of the GFP^+^CD25^+^ CD4SP cell population was largely comparable between Foxp3^GFP^, Foxp3^IRES-GFP^ and BAC-Foxp3^Cre-GFP^ mice (Fig. 2C, bottom).

### Correlating Foxp3 and GFP Expression in Thymus and Periphery

**Figure 3:**
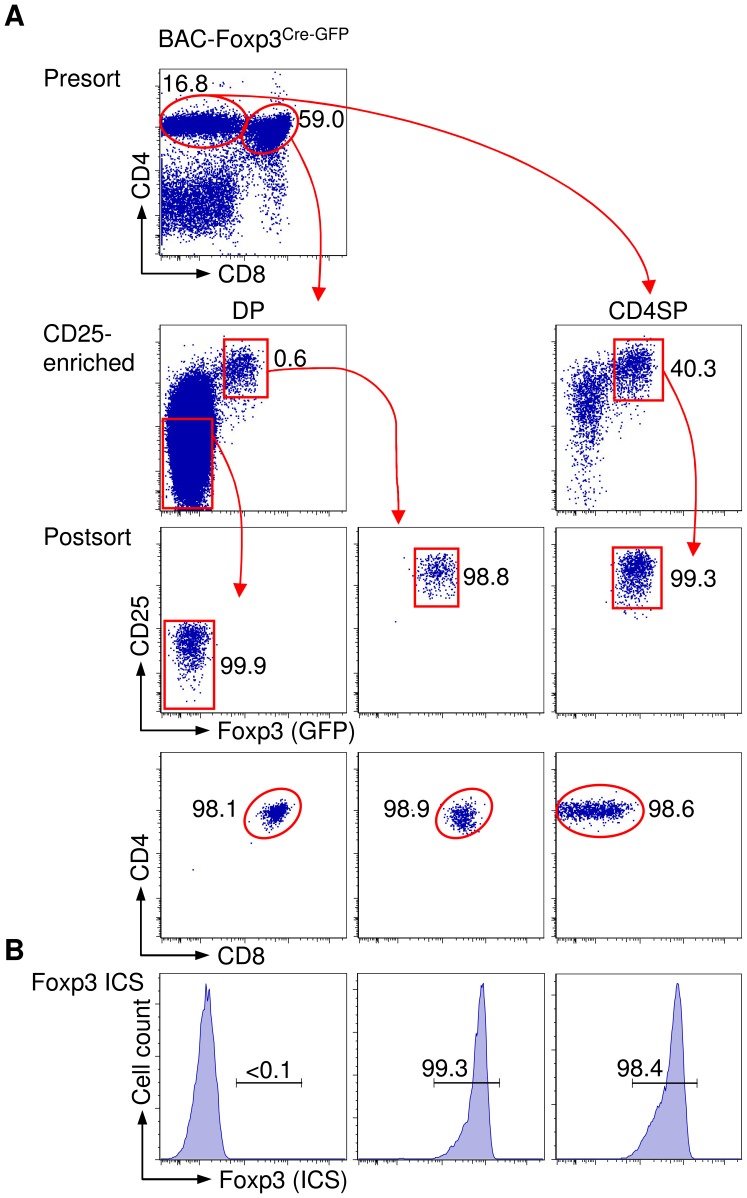
BAC-Foxp3^Cre-GFP^-driven GFP expression faithfully reflects Foxp3 protein expression during thymic Treg cell lineage commitment. Direct assessment of Foxp3 protein expression using mAbs to Foxp3 after flow cytometric isolation of GFP^+^ cells from BAC-Foxp3^Cre-GFP^ mice. (A) Presort analysis of CD25 and GFP expression among gated DP and CD4SP cells from BAC-Foxp3^Cre-GFP^ mice after magnetic bead enrichment of CD25^+^ cells, as well as postsort analysis are depicted, as indicated. CD25^?^GFP^?^ DP cells were included for comparison (left). (B) Foxp3 expression of sorted cells, as revealed by intracellular staining (ICS) with mAbs to Foxp3. Lines with arrowheads illustrate the gating scheme. Numbers in dot plots and histograms indicate percentages of cells in the respective quadrant or gate.

**Figure 4:**
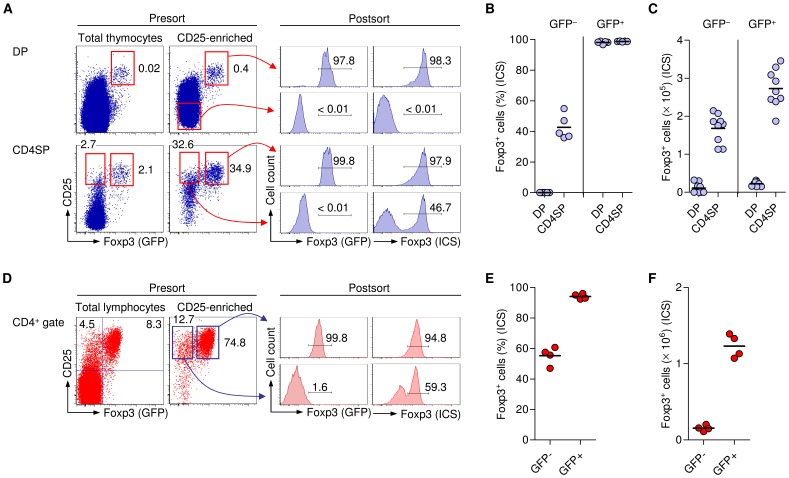
Infidelity of BAC-Foxp3^Cre-GFP^-dependent GFP expression. (**A-C**) Tracking Foxp3^+^ cells that lack GFP expression during thymic Treg cell lineage commitment. (**A**) Representative dot plots depict presort analysis of CD25 and GFP expression among gated DP and CD4SP thymocyte subsets from six-week-old BAC-Foxp3^Cre-GFP^ mice before and after magnetic bead enrichment of CD25^+^ cells, as indicated. Histograms show postsort analysis of GFP expression (left) and Foxp3 expression (right), as revealed by Foxp3 ICS, among indicated postsort populations. (**B**) Percentages and (**C**) numbers of Foxp3^+^ cells (ICS) among GFP^−^ and GFP^+^ cells that had been sorted from DP and CD4SP thymocyte compartments. (**D-F**) Tracking Foxp3^+^ Treg cells that lack GFP expression in peripheral lymphoid tissues. (**D**) Representative dot plots depict presort analysis of CD25 and GFP expression among gated CD4^+^ T cells from LNs before and after magnetic bead enrichment of CD25^+^ cells. Histograms show postsort analysis of GFP expression (left) and Foxp3 expression, as revealed by Foxp3 ICS (right), among indicated postsort populations. (**E**) Percentages and (**F**) numbers of Foxp3-expressing (ICS) CD4^+^CD25^+^ T cells among GFP^−^ and GFP^+^ cells that had been isolated by flow cytometry from pooled scLNs of BAC-Foxp3^Cre-GFP^ mice. All mice were six weeks old. Lines with arrowheads in dot plots illustrate the gating scheme. Numbers in dot plots and histograms indicate percentages of cells in the respective quadrant or gate. Dots and horizontal lines in graphs indicate individual mice and mean values, respectively.

We isolated highly pure populations of GFP-expressing CD25^+^ DP and CD25^+^ CD4SP thymocytes from BAC-Foxp3^Cre-GFP^ mice (Fig. 3A) to directly assess Foxp3 protein expression by ICS with mAbs to Foxp3 (Foxp3 ICS). This approach demonstrated that GFP expression in both DP and CD4SP thymocytes from BAC-Foxp3^Cre-GFP^ mice faithfully reflected Foxp3 expression (Fig. 3B, Fig. 4A,B). Similar results were obtained with GFP^+^ DP thymocytes from Foxp3^IRES-GFP^ and BAC-Foxp3^DTR-GFP^ mice (data not shown). However, up to 50% of BAC-Foxp3^Cre-GFP^ transgenic CD25^+^ CD4SP thymocytes lacking GFP expression exhibited expression of Foxp3 (Fig. 4A,B), corresponding to up to 38.1±7.1% of the overall pool of Foxp3^+^ thymocytes in BAC-Foxp3^Cre-GFP^ mice, as calculated based on absolute numbers of GFP^?^ and GFP^+^ cells among Foxp3^+^CD4^+^CD25^+^ T cells (Fig. 4C). Similarly, although GFP expression in CD4^+^CD25^+^ T cells from peripheral lymphoid organs of BAC-Foxp3^Cre-GFP^ mice closely reflected the expression of Foxp3 protein (Fig. 4D,E), we consistently observed that up to 60% of peripheral CD4^+^CD25^+^GFP^?^ T cells expressed Foxp3 protein (Fig. 4D,E). Based on the numbers of GFP^?^ and GFP^+^ cells depicted in Fig. 4F, we estimated the relative contribution of Foxp3^+^GFP^?^ cells to the overall pool of peripheral Foxp3^+^ Treg cells in adult BAC-Foxp3^Cre-GFP^ mice to be 11.3±2.7%.

### Fate Mapping of Foxp3^+^GFP^−^ Treg Cells

**Figure 5:**
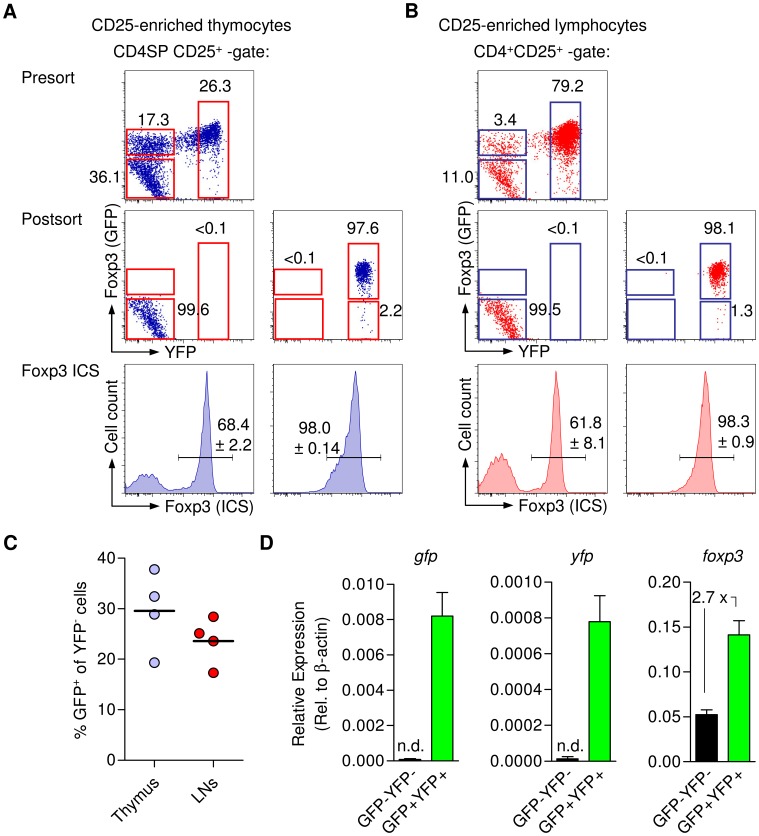
Genetic lineage tracing of Foxp3^+^ Treg cells in BAC-Foxp3^Cre-GFP^ × R26Y mice. Analysis of Foxp3 expression by Foxp3 ICS, in FACS purified GFP^−^YFP^−^ and GFP^−^/GFP^+^YFP^+^ populations among (**A**) CD25^+^ CD4SP thymocytes and (**B**) CD4^+^CD25^+^ T cells from LNs of BAC-Foxp3^Cre-GFP^ × R26Y mice. Representative presort analysis of GFP and YFP expression among gated CD4^+^CD25^+^ cells and postsort analysis are shown as dot plots. Histograms depict Foxp3 expression (ICS) in sorted GFP^−^YFP^−^ (left) and GFP^−^/GFP^+^YFP^+^ (right) cells. Numbers in dot plots and histograms indicate percentages of cells in the respective quadrant or gate. Data are representative of three independent experiments including at least three mice. (**C**) Percentages of GFP^+^ cells among *ex vivo* populations of YFP^−^CD25^+^ CD4SP thymocytes (left) and peripheral YFP^−^CD4^+^CD25^+^ T cells (right). (**D**) mRNA expression of GFP, YFP and Foxp3 was determined by real-time RT-PCR in sorted GFP^−^YFP^−^ and GFP^−^/GFP^+^YFP^+^ cells presented in (**A and B**).

Possible reasons underlying the presence of significant Foxp3^+^GFP^−^ T cell populations in both thymus and peripheral lymphoid tissues of BAC-Foxp3^Cre-GFP^ mice include delayed up-regulation of GFP expression that lags behind up-regulation of Foxp3 expression in developing Treg cells, the failure to induce Foxp3 BAC transcriptional activity in some Foxp3^+^ Treg cell lineage-committed cells, and the down-regulation of Foxp3 BAC expression in mature Foxp3^+^ Treg cells. In an initial attempt to address these issues, we sought to track Foxp3 and GFP expression in BAC-Foxp3^Cre-GFP^ mice that additionally expressed YFP under the control of the Rosa26 promoter only after excision of a loxP-flanked STOP cassette (R26Y) [39]. To this end, YFP-expressing cells among CD25^+^ CD4SP thymocytes (Fig. 5A) and peripheral CD4^+^CD25^+^ Treg cells (Fig. 5B) were FACS purified from BAC-Foxp3^Cre-GFP^ × R26Y mice, irrespective of their GFP expression status. Postsort analysis revealed that the vast majority of YFP^+^ cells from thymus (97.0±1.28%) and periphery (97.9±0.21%) exhibited co-expression of GFP, suggesting a minor contribution of transcriptional down-regulation of the BAC-Foxp3^Cre-GFP^ transgene in initially Foxp3^+^GFP^+^ T cells to the populations of Foxp3^+^GFP^?^ cells (Fig. 4). As expected, Foxp3 ICS revealed that YFP expression closely correlated with the expression of Foxp3 protein (Fig. 5A,B) in both CD25^+^ CD4SP thymocytes (98.0±0.14%) and peripheral CD4^+^CD25^+^ Treg cells (98.3±0.9%). In addition, BAC-Foxp3^Cre-GFP^ × R26Y mice allowed us to track sizable YFP^?^ populations among CD25^+^ CD4SP thymocytes and peripheral CD4^+^CD25^+^ T cells, which were found to be either GFP^?^ or GFP^+^ (Fig. 5A and B, presort; Fig. 5C). It appears reasonable to speculate that thymic and peripheral GFP^+^YFP^?^ T cells represent developing Foxp3^+^ Treg cells that, after having successfully up-regulated expression of both Foxp3 protein and the Cre recombinase-GFP fusion protein, are in the process of Cre-mediated excision of the loxP-flanked STOP cassette and subsequent up-regulation of YFP expression. In fact, initially GFP^+^YFP^?^ cells appear to gradually increase YFP expression until reaching maximum levels (see presort analysis, Fig. 5A and B). Unexpectedly, Foxp3 ICS revealed that the majority of GFP^?^YFP^?^ cells among CD25^+^ CD4SP thymocytes (68.4±2.2%; Fig. 5A, bottom) and peripheral CD4^+^CD25^+^ cells (61.8±8.1%; Fig. 5B, bottom) exhibited Foxp3 protein expression. In addition to the absence of GFP and YFP protein, *gfp* and *yfp* mRNA expression was found to be below the level of detection in peripheral GFP^?^YFP^?^CD4^+^CD25^+^ T cells of BAC-Foxp3^Cre-GFP^ × R26Y mice, whereas expression of *Foxp3* mRNA was readily detectable (Fig. 5D). These data are consistent with our interpretation that the Foxp3 BAC transgene is transcriptionally inactive in the GFP^?^YFP^?^ subset among peripheral CD4^+^CD25^+^Foxp3^+^ T cells from BAC-Foxp3^Cre-GFP^ × R26Y mice.

## Discussion

Here, we have extended the previous observation of exceedingly low numbers of GFP^+^ DP thymocytes in Foxp3^GFP^ mice [12] and provided evidence that Foxp3^GFP^ mice exhibit an approximately ten-fold reduction in the size of the Foxp3^+^ DP cell population, as compared with Foxp3^Cre-GFP^ mice. In fact, numbers of Foxp3^+^ DP cells were found to be close to the level of detection in both C57BL/6 and BALB/c Foxp3^GFP^ mice, suggesting a minor influence of the mouse genetic background on the near complete absence of Foxp3^+^GFP^+^ DP cells. Considering that the propensity of Foxp3^?^ DP cells to form doublets with Foxp3^+^ CD4SP cells poses a risk of overestimating numbers of Foxp3^+^ DP cells [12], it is important to emphasize that, in the present study, magnetic bead enrichment and stringent gating criteria for FACS purification of GFP-expressing cells were employed to mechanically disrupt doublets for enumeration of DP thymocytes that are truly Foxp3^+^. Our interpretation that the existence of a population of GFP^+^ DP cells in the thymus of BAC-Foxp3^Cre-GFP^ mice is physiological rather than a reflection of untimely aberrant expression of the BAC transgene is strongly supported by an overwhelming correlation between GFP and Foxp3 expression, as revealed by ICS with mAbs to Foxp3 in highly pure populations of Foxp3^Cre-GFP^ transgenic GFP^+^ DP thymocytes.

It is of interest to note that, in addition to aberrations in thymic Treg cell induction, our unpublished observations additionally indicated that naïve CD4^+^ T cells (CD62L^high^CD25^–^GFP^–^) from Foxp3^GFP^ mice (58.8±5.8%) are somewhat impaired in their heir capacity to undergo TGF-β-mediated up-regulation of Foxp3 expression *in vitro*, as compared to cells from BAC-Foxp3^Cre-GFP^ (82.6±2.9%) and BAC-Foxp3^DTR-GFP^ (79.4±3.9%) mice. In fact, it is only recently that Foxp3^GFP^ mice have been shown to profoundly accelerate spontaneous autoimmune diabetes on the NOD genetic background [37], [38] but to alleviate arthritis symptoms in the K/BxN model [38]. This divergent immune regulation in Foxp3^GFP^ mice could be attributed to transcriptional dysregulation and defective extrathymic Treg cell induction, probably due to alterations in molecular interactions of the N-terminal GFP-Foxp3 fusion protein with specific transcriptional cofactors [37], [38]. Additional studies are clearly warranted to address a putative contribution of aberrant thymic Treg cell development, as revealed by the relative deficiency of Foxp3^+^ DP cells, to the divergent immune regulation in Foxp3^GFP^ mice [37], [38].

The absence of YFP expression in Foxp3^+^GFP^+^ T cells from thymus and peripheral lymphoid organs of BAC-Foxp3^Cre-GFP^ × R26Y mice is likely to mark a late Treg cell developmental stage, on the brink of up-regulating YFP expression, after Cre-mediated excision of the loxP-flanked STOP cassette. However, it remains to be determined whether such GFP^+^YFP^?^ Treg cells in the periphery originate from intrathymic lineage commitment, having entered peripheral lymphoid tissues prior to the up-regulation of detectable YFP levels, or whether Treg cell lineage commitment has occurred outside the thymus in peripheral lymphoid tissues [27].

Additionally, this study provided evidence that up to 15% of the overall pool of peripheral Foxp3^+^ Treg cells in adult BAC-Foxp3^Cre-GFP^ × R26Y mice exhibit a GFP^?^YFP^?^ cell phenotype. Keeping in mind that the Cre recombinase is expressed as a GFP fusion protein, the existence of a sizable population of Foxp3^+^GFP^?^ Treg cells in secondary lymphoid organs of BAC-Foxp3^Cre-GFP^ mice seems difficult to reconcile with the observation that Dicer^lox/lox^ × BAC-Foxp3^Cre-GFP^ mice develop fatal systemic autoimmune disease [35], resembling the phenotype of Foxp3-deficient mice in many aspects, albeit with delayed mortality kinetics. Notably, in Dicer^lox/lox^ × BAC-Foxp3^Cre-GFP^ mice, deletion of Dicer at the time of Foxp3 and GFP induction (i.e. after full commitment to the Treg cell lineage) had only minimal effects on thymic Treg cell development and peripheral export. In the periphery, Dicer deletion resulted in dysfunctional Foxp3^+^GFP^+^ Treg cells that acquired a T helper cell phenotype, including effector cytokine production [35]. Conceivably, in addition to exacerbated T effector cell activation due to reduced numbers of functional Treg cells, autoimmune disease in Dicer^lox/lox^ × BAC-Foxp3^Cre-GFP^ mice is likely to be driven by self-reactive, initially normal but unstable Treg cells that, as a consequence of Dicer deletion, dedifferentiate into autoimmune T effector cells, overwhelming the population of functional Foxp3^+^GFP^?^ Treg cells that reside in the periphery.

At present, we can only speculate on the mechanisms underlying the absence of Foxp3 BAC transcriptional activity in the peripheral population of Foxp3^+^GFP^?^YFP^?^ cells among CD4^+^CD25^+^Foxp3^+^ Treg cells in BAC-Foxp3^Cre-GFP^ × R26Y mice. As exemplified by the overwhelming co-expression of Foxp3 protein in GFP-expressing T cells from Foxp3^Cre-GFP^ (Fig. 3 and 4) and Foxp3^DTR-GFP^ mice (data not shown), the use of large genomic fragments for BAC transgenesis generally ensures faithful expression of the transgene under the control of most, if not all regulatory regions of a gene in its genomic configuration [40]. While integration of multiple copies results in high levels of expression of the BAC transgene, the endogenous mouse gene is not altered and the integration site in the host genome is likely to have limited impact on BAC transgenic expression. However, BAC expression faithfully mimicking the natural expression of the respective endogenous gene will critically depend on the structural integrity of the BAC transgene. These considerations point towards the possibility that the deficiency of peripheral Foxp3^+^GFP^?^YFP^?^ cells among CD4^+^CD25^+^ Treg cells in BAC-Foxp3^Cre-GFP^ × R26Y mice to up-regulate Foxp3 BAC expression upon Treg cell lineage commitment may rely on altered transcription regulation of the Foxp3 BAC gene due to partial integration of the BAC transgene, the inserted GFP encoding gene or both. In any case, the identification of the exact mechanisms underlying differential Foxp3 BAC expression in BAC-Foxp3^Cre-GFP^ mice might be a worthwhile task, as such studies promise to provide novel insight into the critical role of DNA regulatory elements in *Foxp3* gene regulation during Treg cell development.
